# Mammographic Screening in the Occupied Palestinian Territory: A Critical Analysis of Its Promotion, Claimed Benefits, and Safety in Palestinian Health Research

**DOI:** 10.1200/JGO.19.00383

**Published:** 2020-11-18

**Authors:** Shaymaa AlWaheidi, Klim McPherson, Iain Chalmers, Richard Sullivan, Elizabeth A. Davies

**Affiliations:** ^1^ Cancer Epidemiology, Population and Global Health, King's College London, London, United Kingdom; ^2^ Public Health Epidemiology, Nuffield Department of Primary Health Care Research and New College, University of Oxford, Oxford, United Kingdom; ^3^ Centre for Evidence-Based Medicine, Nuffield Department of Primary Care Health Sciences, University of Oxford, Oxford, United Kingdom; ^4^ Institute of Cancer Policy, Kings Health Partners Comprehensive Cancer Centre, King's College London, London, United Kingdom

## Abstract

**PURPOSE:**

To critically review the evidence and opinions expressed about mammographic screening (MS) in research reports on breast cancer in the occupied Palestinian territory (oPt) and to assess whether benefits and harms in MS are presented in a balanced way.

**METHODS:**

Searches of PubMed, Cochrane, MEDLINE, EMBASE, CINAHL, and gray literature identified 14 eligible research reports relating to the oPt. We reviewed these documents and then used a thematic analysis to describe and analyze the evidence and the opinions about MS expressed in them.

**RESULTS:**

All 14 research reports mentioned that MS would improve survival rates in the oPt. Only three gave information on major harmful effects, and only two emphasized that MS must be accompanied by effective treatment to have any beneficial effects on population mortality. There was no consistency in the recommended frequency of MS.

**CONCLUSION:**

Most information presented by Palestinian health researchers was selective and failed to address the important established harms of MS. Thus, calls to support MS in the oPt are not based on a measured discussion of the risks and benefits for women or grounded in the systemic readiness of health care necessary for its effectiveness. As long as diagnostic and treatment facilities remain deficient, screening cannot lead to reduced mortality from breast cancer.

## INTRODUCTION

Since its introduction, the relative harms and benefits of mammographic screening (MS) have been the subject of continuing debate. A balanced evidence-based presentation of both the benefits and harms of screening to women will always be required to avoid confusion, misleading information, and genuine harm. Also, regardless of a pro- or anti-MS policy stance, there is general agreement that improvements in early presentation and diagnosis, and evidence-based combined-modality treatment (surgery, radiotherapy, and systemic drug therapy) should occur before any decision is made to initiate population MS or not.^1^ Despite the lack of such essential prerequisites, MS has been promoted in many low- and middle-income countries despite a lack of evidence in these contexts, including data on system-specific cost effectiveness or parallel improvements in diagnostic and treatment facilities.^2,3^

**CONTEXT**

**Key Objective**
Are the benefits and harms of mammographic screening presented in a balanced way in Palestinian health research?
**Knowledge Generated**
Most Palestinian health research studies included in our review encourage women to undergo mammographic screening without informing them about its important harmful effects, such as overdiagnosis and subsequent overtreatment. They also publish conflicting recommendations on the frequency of mammographic screening.
**Relevance**
Because sound policies rest on good information, benefits and harms in mammographic screening need to be assessed and presented in a balanced way. Information on this intervention should also reflect the level of scientific uncertainty, allowing women to understand the possible benefits and harms of screening and reach a decision by themselves.

In the occupied Palestinian territory (oPt), a politically volatile low-income country, the focus on MS and the provision of mammography machines has involved the Palestinian government, as well as international humanitarian and local nongovernmental organizations. In its 2017-2022 national health strategy document,^4^ the Palestinian government states that providing more MS services is one of its top priorities. There are 19 mammographic machines in the West Bank and 20 mammographic machines in Gaza (19 in the government sector, with the remainder in the private and nongovernmental sector).^5^ However, 90% of these machines are either nonfunctioning or underutilized, partly because there are no well-trained personnel to operate them and interpret mammographic films or because of frequent strikes in the health system because of wages having been withheld.^5^ Despite this, many health facilities are devoting more of their budgets to purchasing mammographic machines. In September 2017, the government celebrated the inauguration of a digital mammographic machine in a government clinic in Gaza at a cost of USD 130,000.^6^ This was followed by the purchase of a three-dimensional mammographic machine in the first breast cancer unit in Palestine in the West Bank in December 2018,^7^ with these purchases being facilitated by donations from international organizations.

As more MS machines are being purchased, the call for Palestinian women to undergo screening becomes louder. Every year in October, the oPt turns pink with street banners and campaigns by international and local organizations encouraging women to undergo screening because early detection saves lives, mixing two different issues, that is, population-based MS screening and early detection. There is no doubt that these campaigns can be successful in raising awareness of breast cancer symptoms and early detection, and in tackling disease-related stigma and fatalism, but they tend not to be well targeted and are subject to biased presentation of information regarding the relative harms and benefits of MS. The only available awareness information on MS at the Palestinian Ministry of Health-Ramallah website simply tells women 40-50 years of age to get screened every other year and every year for those over the age of 50 because “MS helps to detect breast cancer in an early stage when treatment is most successful. It is quick, safe, does not cause pain, and available for free in governmental facilities.”^8^ The situation is no different at the Palestinian Ministry of Health-Gaza website, which also exaggerates the benefits but neglects to highlight the harms and simply encourages women to undergo MS.^9^

The country’s policies and campaigns for MS do not mention the nature of the evidence that has been used, nor whether the campaigns relied on local evidence in their recommendations. Therefore, in this article, we critically review the evidence reported about MS in reports of research on breast cancer in the oPt to assess whether local campaigns could be a reflection of the evidence available in Palestinian health research. By reviewing the evidence, we aimed to understand whether Palestinian health research presents MS benefits and harms in a balanced way or whether it tends to exaggerate benefits and fails to highlight the harms involved, as does worldwide research.^10^ We also aimed to understand whether the opinions expressed about MS in Palestinian health research are in line with the country’s local policy making and practice.

## METHODS

### Research Question

The research question for this review was: Are the benefits and harms of MS presented in a balanced way in Palestinian health research? The elements of the PICO question (patient/population, intervention/indicator, compare/control, outcome, time/type of study, or question) included in this review were:

- population of interest: Palestinian health research documenting any MS benefit or harm, or both;
- intervention: MS;
- comparator: no comparison group was studied; and,
- outcomes: the primary outcome was evidence on MS benefits and harms documented in Palestinian health research on breast cancer.

### Search Strategy and Selection Criteria

We included all Palestinian health research articles on MS that described a benefit or a harm, or both, and were published in Arabic or English until the end of December 2019. We excluded reports that were abstracts or conference proceedings, studies that focused on palliative care for women with breast cancer, studies that concentrated exclusively on breast cancer survival statistics, and reports that did not provide any evidence on MS. A three-step search strategy without language restrictions was used by S.A.W. to identify potentially relevant research reports. First, the following five academic databases were searched: PubMed, CINAHL, EMBASE, MEDLINE, and Cochrane Central Register of Controlled Trials. The search terms included: mammography, screening, early detection, mammogram, breast cancer, breast tumour, women with cancer, malignant, carcinoma, neoplasm, Gaza, East Jerusalem, Palestine, West Bank, Palestinian women, and occupied Palestinian territory. Second, reference lists of reports retrieved in the first step were screened for potentially relevant research reports. Third, additional online platforms, including Google searches for gray literature, EThOS (Electronic Theses Online Service), and the Lancet-Palestinian Health Alliance conference abstracts were searched. Finally, after a review of research reports, some researchers were contacted to obtain relevant unpublished reports. If several full-text reports were found for a single study, the report that was published in a scientific journal was the one that was selected for additional analysis in our review, and we only used master’s theses when there was no alternative. Master’s theses were included in our review because the oPt is a low-income country that lacks an academic community to support research resources necessary for articles to be commonly published in scientific journals. We also hypothesized that the output of these theses would be relevant because the opinions about MS expressed in them were likely to reflect current practice if the authors had been employed by a health care facility at the time the theses were published. The process of screening for selection of the included studies was performed by full-text review by S.A.W., and then the results of the screening were reviewed by E.A.D. Data from the selected research reports were read and discussed by S.A.W., K.M., I.C., and E.A.D. Data were extracted by two reviewers: E.A.D. extracted data from the reports, and S.A.W. crosschecked these for accuracy. Five main categories of data were extracted for each study: author names and affiliation at the time of publication, characteristics of the sample, methodologic approach, results, and the evidence cited about MS.

### Data Analysis

A thematic analysis was performed by S.A.W., as described by Clarke and Braun^11^ to document the source of authors’ opinions about MS. S.A.W. read each report several times to make notes about the opinions expressed and then assessed whether the information presented in the research reports gave a balanced account of the possible benefits and harms of MS. The results were checked by E.A.D., discussed with S.A.W., and reviewed by I.C., and any disagreements were settled by discussion. To facilitate the process, a data sheet that contained the same 16 information items as in the study published by Jørgensen and Gøtzsche^12^ was used to report the information on benefits and harms from MS in the included studies.^12^

## RESULTS

Of the 37 research reports identified, three were excluded because they were duplicates, leaving 34 reports that we screened for potential relevance (Fig 2). Among these, we identified 14 full-text research reports that provided data relevant to MS in the oPt (Table 1; Appendix). Of these, six studies provided data on factors underlying the low use of MS (Jaddallah A: Evaluation of mammogram services in the Gaza Strip Governorates [master’s thesis]. Al-Quds University, Palestine, 2016),^13-17,^ three documented the characteristics of women at diagnosis and assessed which features were associated with length of survival (Al-Agha L: Survival determinants of breast cancer cases in Gaza Governorates [master’s thesis]. Al-Quds University, Palestine, 2014),^18,19^ one reported factors affecting stage of diagnosis among women with breast cancer,^20^ one focused on factors affecting early presentation among women,^21^ one reported breast cancer detection rates by MS and the quality and completeness of cancer registry data,^22^ one reported risk factors for breast cancer,^23^ and one investigated the characteristics of women who undergo diagnostic mammography and MS (Table 2).^24^ Four themes emerged from the thematic analysis of Palestinian health research on breast cancer. The reports (1) recommend MS and give inconsistent recommendations on its frequency; (2) misinterpret survival statistics and overstate the benefit of MS; (3) ignore the important established harms of MS; and (4) do not seem to be improving in quality over time (Table 3).

**FIG 1:**
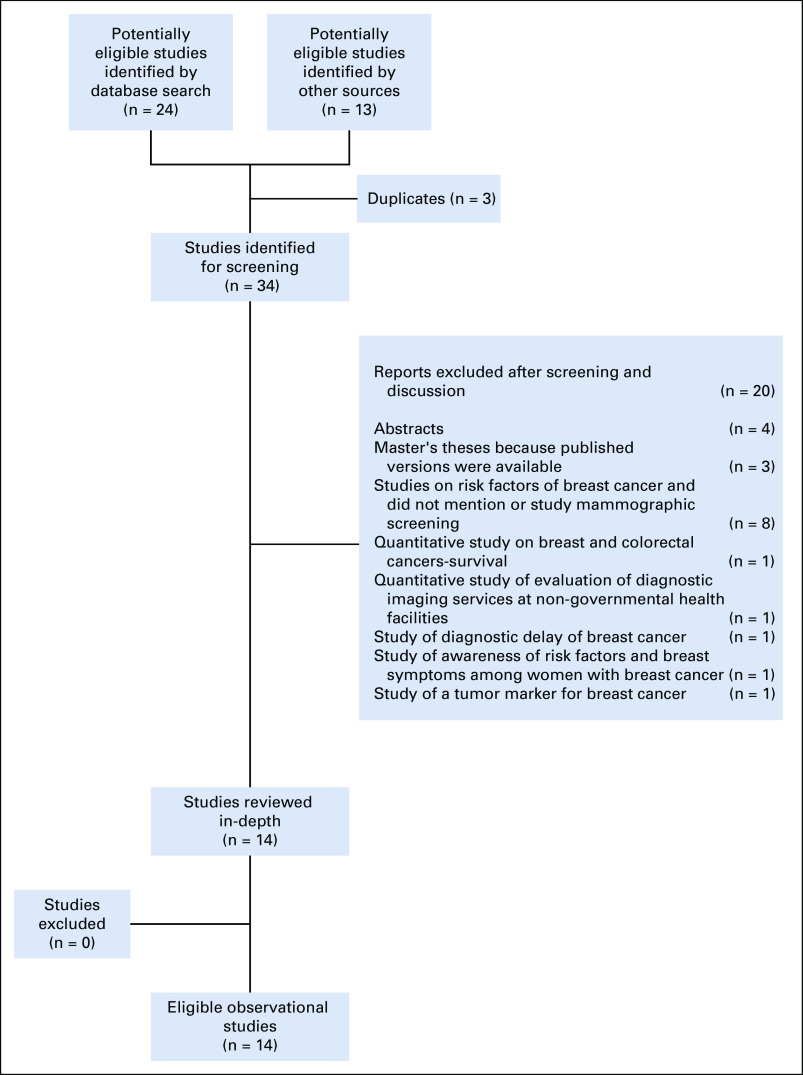
Selection of studies for this review.

**TABLE 1:**
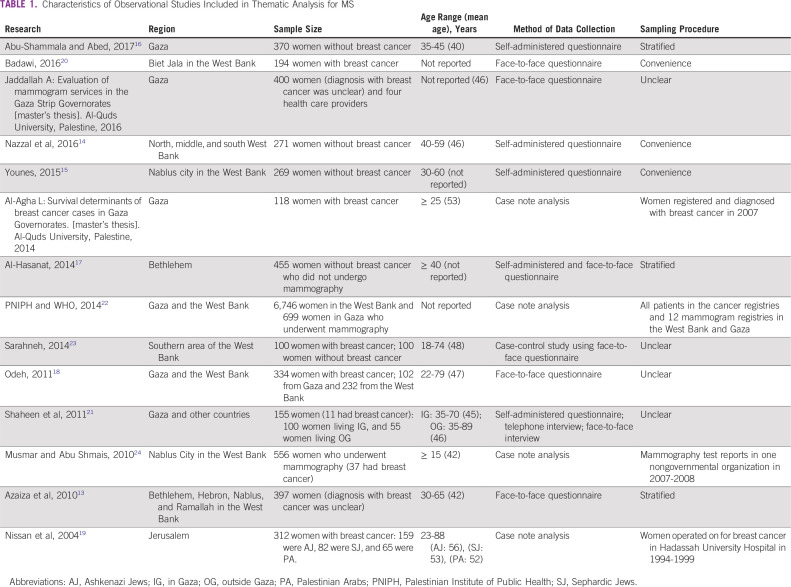
Characteristics of Observational Studies Included in Thematic Analysis for MS

**TABLE 2:**
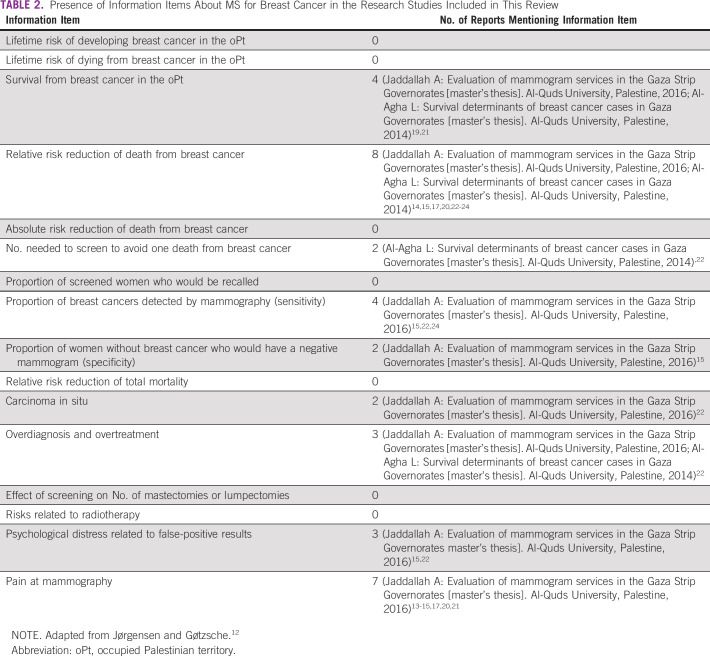
Presence of Information Items About MS for Breast Cancer in the Research Studies Included in This Review

**TABLE 3:**
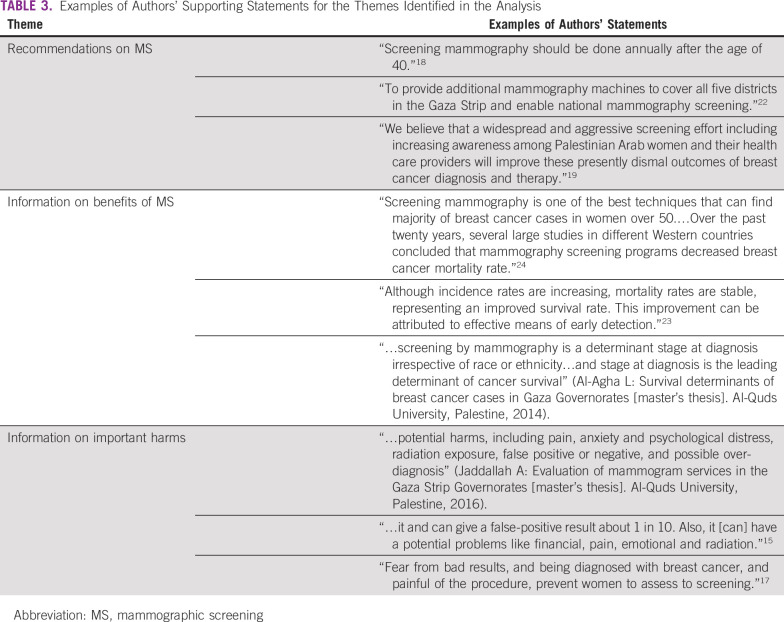
Examples of Authors’ Supporting Statements for the Themes Identified in the Analysis

### Recommendations on MS

Eleven of the 14 reports recommended population MS despite the lack of the necessary infrastructure to provide and maintain high-quality screening programs and follow-up care (Table 4).^14-18,20,22-24^ Data in the research reports revealed inconsistencies in the recommended frequency of MS. Seven reports agreed that MS should be performed annually for all women after the age of 40 years.^13-15,17,18,22,24^ Younes^15^ claimed that the Palestinian Ministry of Health also recommended MS every 3 years for all women 20-30 years of age, but Jaddallah (Evaluation of mammogram services in the Gaza Strip Governorates [master’s thesis]. Al-Quds University, Palestine, 2016) stated that mammograms are not recommended for women under 40 years of age. Badawi^20^ maintained that MS should be performed every 3 years for all women 35-40 years of age, every other year for women aged 40-50 years of age, and annually for those over the age of 50 years. These recommendations were not based on any evidence available at the time of issue.

**TABLE 4:**
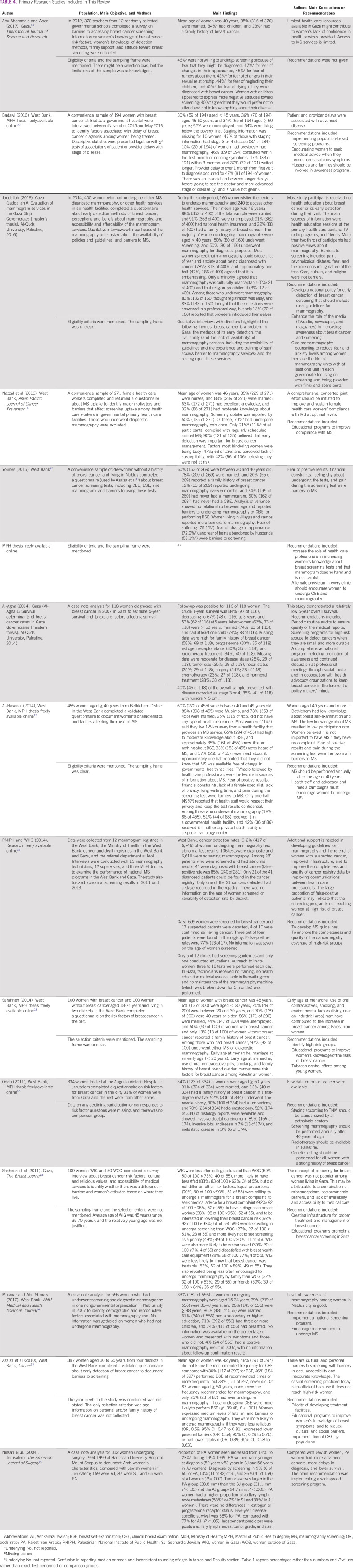
Primary Research Studies Included in This Review

Only two reports emphasized that population MS requires concomitant efficient and effective treatment to have any important effects on population mortality from breast cancer.^13,21^ One other recommended a good surveillance system and early detection through screening for high-risk groups (eg, for women who had already received breast cancer treatment), so that cancers could be detected when they are small and potentially more curable (Al-Agha L: Survival determinants of breast cancer cases in Gaza Governorates [master’s thesis]. Al-Quds University, Palestine, 2014).

### Claimed Benefits of MS

All reports asserted that MS would improve breast cancer survival rates in the oPt. The benefit mentioned most often was a relative reduction in breast cancer mortality, and estimates varied from an 18% to 45% reduction. No report mentioned absolute risk reduction in breast cancer mortality or how many women might benefit. Many of these reports significantly overestimated the benefits plausibly attributable to MS. Nazzal et al^14^ argued that mammography is the only effective breast cancer screening method. One report claimed that MS can detect up to 90% of patients with breast cancer,^15^ whereas another claimed that MS can detect breast cancer 2 years before a lump can be felt.^17^ All reports emphasized possible benefits in a way that would encourage women to undergo MS. Three reports indicated that screening leads to better treatment options,^14,17,23^ and one stated that it contributed to fewer mastectomies.^15^ Of the 14 research reports, only five cited evidence from systematic reviews of MS (Jaddallah A: Evaluation of mammogram services in the Gaza Strip Governorates [master’s thesis]. Al-Quds University, Palestine, 2016; Al-Agha L: Survival determinants of patients with breast cancer in Gaza Governorates [master’s thesis]. Al-Quds University, Palestine, 2014),^13-15,22^ whereas others based their recommendations on individual studies. This led to a less balanced perspective in their conclusions.

### Information on Important Harms

Of the 14 reports, only one published and two unpublished reports mentioned important harmful consequences related to cancers that may never progress, including overdiagnosis and overtreatment (Jaddallah A: Evaluation of mammogram services in the Gaza Strip Governorates [master’s thesis]. Al-Quds University, Palestine, 2016; Al-Agha L: Survival determinants of breast cancer cases in Gaza Governorates [master’s thesis]. Al-Quds University, Palestine, 2014; (Table 2).^22^ Three reports mentioned anxiety or psychological distress related to false-positive results (Jaddallah A: Evaluation of mammogram services in the Gaza Strip Governorates [master’s thesis]. Al-Quds University, Palestine, 2016).^15,22^ One report stated that the rate of false-positive results among women undergoing MS is 85.4% (240 of 281) in the West Bank and 76.5% (13 of 17) in Gaza,^20^ but the reasons for these high rates were not considered or investigated. They may indicate reporting issues or that the screening program was not reaching women at high risk for breast cancer. Seven reports mentioned the potential pain during the mammographic procedure (Jaddallah A: Evaluation of mammogram services in the Gaza Strip Governorates [master’s thesis]. Al-Quds University, Palestine, 2014).^13-15,17,20,21^ The other five did not mention any possible harmful effects and referred only to the claimed positive effects. None provided estimates of the cost effectiveness of the intervention in a low-income country such as the oPt.

### Author Affiliation at the Year of Publication

Of the 14 reports, seven were master’s theses, mainly for public health programs (with five of them from the same university; Jaddallah A: Evaluation of mammogram services in the Gaza Strip Governorates [master’s thesis]. Al-Quds University, Palestine, 2016; Al-Agha L: Survival determinants of breast cancer cases in Gaza Governorates [master’s thesis]. Al-Quds University, Palestine, 2014).^15,17,18,20,23^ In all theses, it was not clear whether authors were employed. Three reports were from authors working at higher education institutions,^13,19,21^ and one report was published by a governmental research organization in support of an international body.^22^ We could not establish a possible relationship between author affiliation and the quality of evidence cited on MS, nor could we detect any significant improvement in the quality of evidence cited on MS in relation to the year of publication. We would expect that the quality for evidence reported would improve over time, but that does not appear to be the case.

## DISCUSSION

### Summary of Main Findings

Despite deficiencies in cancer services, the Palestinian Ministry of Health has promoted and prioritized MS. As a result, many health facilities are devoting more of their budgets to purchasing mammographic machines, seemingly with limited analysis of their cost effectiveness or the need for parallel improvements in diagnostic and treatment facilities. Facilities also encourage women to undergo MS without informing them about its potential harmful effects. We critically reviewed the opinions expressed about MS in reports of research on breast cancer in the oPt to assess whether the country’s policies on MS are guided by the evidence available in Palestinian health research and whether MS benefits and harms are presented in a balanced way. Our review makes clear that the authors of most research reports on MS for breast cancer in the oPt see it as their duty to promote the intervention, regardless of the lack of evidence or logic for this. Many reports significantly overestimated the reduction in breast cancer deaths plausibly attributable to MS, even in high-functioning, high-income health care systems. The established harmful consequences of MS, which are overdiagnosis and subsequent overtreatment, were mentioned by three reports only. The fact that many reports included were master’s degree theses is not surprising. Along with many other low- and middle-income countries, the oPt lacks sufficient health research capacity, with most studies conducted by single researchers.

### Comparison With Findings From Previous Research

To our knowledge, this analysis is the first to consider the promotion, claimed benefits, and safety of MS research in the oPt. It provides sufficient evidence that the interests of Palestinian women are not being well served. Our findings are consistent with previous evidence in other countries, suggesting that invitations to MS tend to exaggerate benefits and omit harms while encouraging women to undergo MS.^10,12,25,26^

**FIG 2:**
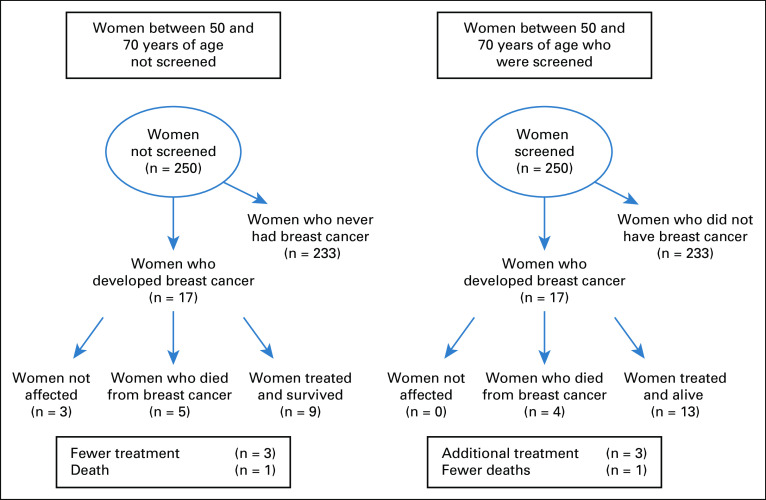
Tree diagram showing the consequences for 250 women undergoing mammographic screening presented by David Spiegelhalter, OBE, FRS (developed based on Informed Choice About Cancer Screening at King’s Health Partners, 2012).

Up-to-date evidence from systematic reviews is available to help develop a balanced presentation of benefits and harms in MS,^27-31^ and the use of such evidence could help improve the quality of Palestinian health research and policy on breast cancer in the future. Researchers should explain benefits and harms in a transparent and balanced way, as illustrated in the information leaflet developed by the Nordic Cochrane Centre or as presented in Figure 2 by Spiegelhalter.^32,33^

### Strengths and Weaknesses of the Study

To our knowledge, this review is the first to consider the promotion, claimed benefits, and safety of MS in the oPt. In doing so, we have considered both the research evidence presented and a thematic analysis of opinions expressed within it. Although our search strategy has located many relevant studies, many of the research reports included were master’s degree theses, and the lack of basic information about most aspects of breast cancer in the oPt inevitably imposed limitations on our review.

### Implications for Practice, Policy, and Future Research

Palestinian women with breast cancer are usually diagnosed at an advanced stage, and limited resources mean that early detection, diagnosis, and treatment cannot be efficiently promoted. There is little routine data in Gaza and the West Bank available on breast cancer with which to estimate the trends in mortality, but the extant data reveal that the 5-year survival rate after the diagnosis of breast cancer is considerably low.^34^ Poor survival for women with breast cancer in the oPt may reflect at least two factors: first, most women with breast symptoms present to the health services with advanced-stage disease (Al-Agha L: Survival determinants of breast cancer cases in Gaza Governorates [master’s thesis]. Al-Quds University, Palestine, 2014)^21^; second, facilities for diagnosis and treatment are inadequate and fragmented, with no clear referral pathways established among providers. Radical mastectomy is widely used,^5^ partly because radiotherapy is not available in Gaza and the West Bank but only provided in East Jerusalem. Palestinian authors were aware of the inadequate diagnostic and treatment services for breast cancer in the oPt. Yet, there is a big mismatch between the strength of support of MS and the lack of support for basic diagnostic and treatment facilities for women with breast cancer in the oPt.

As Bywaters and Knox^35^ pointed out more than 40 years ago, MS cannot be of any benefit unless it is accompanied by effective and specific diagnosis and treatment pathways. Because sound policies rest on good information, possible benefits and harms in MS need to be assessed and presented in a balanced way. Information on this intervention should also reflect the level of scientific uncertainty, allowing women to understand the possible benefits and harms of screening and reach a decision by themselves.

## APPENDIX

### List of Excluded Studies

Four abstracts:

Al-Ramahi R, Nazzal D, Mstafa D, et al: Evaluation of types and treatment protocols for breast cancer among Palestinian women: A retrospective study. *Lancet* 393:S42, 2019(suppl 1).

Nabaa’ HA, Shelleh N: Barriers preventing Palestinian women from having a mammogram: A qualitative study. *Lancet* 391:S16, 2018 (suppl 2).

Lafi MA, Shaheen R: Needs assessment of breast health care in the Gaza Strip. *J Emerg Med Trauma Acute Care* 2016:83, 2016.

Lahham A, ALMasri H, Kameel S: Estimation of female radiation doses and breast cancer risk from chest CT examinations. *Radiat Prot Dosimetry* 179: 303-309, 2018.

Three master’s theses because published versions were available:

Abu-Shammala B: Breast cancer screening among female school teachers Gaza City [master’s thesis]. Al-Quds University, Palestine, 2012.

Abu Shmais FB: Use of mammography test pattern and percentage of breast cancer detected in Nablus District [master’s thesis]. Al-Quds University, Palestine, 2010. https://scholar.najah.edu/sites/default/files/all-thesis/use_of_mammography_test_pattern_and_percentage_of_breast_cancer_detected_in_nablus_district.pdf.

Al Shiekh SS: Evaluation of the used diagnostic imaging methods for breast cancer in Gaza Governorates [master’s thesis]. Al-Quds University, Palestine, 2018. https://dspace.alquds.edu/bitstream/handle/20.500.12213/2076/MT_2018_21511018_8081.pdf?sequence=1&isAllowed=y.

Eight studies of risk factors of breast cancer or did not mention any benefit or harm of mammographic screening:

Darweesh A: Risk factors of breast cancer among Palestinian women in North West Bank [master’s thesis]. An-Najah National University, Nabulus, Palestine, 2009.

Eljedi A, Nofal M: Health-related quality of life and its influencing factors among breast cancer patients in Palestine. *J Womens Health Issues Care* 3:5, 2014.

Ghrayeb F, Rimawz O, Nimer A: Knowledge of breast cancer and its risk factors among Al-Quds University students in Palestine. *Int J Res Med Sci* 6(11):3504-3508, 2018.

Kariri M, Jalambo MO, Kanou B, et al: Risk factors for breast cancer in Gaza Strip, Palestine: A case control study. *Clin Nutr Res* 6:161-171, 2017.

Khateeb I: Breast cancer risk among patients with diabetes attending Beit-Jala Governmental Hospital [master’s thesis]. Al-Quds University, Palestine, 2018.

Sarahneh A: Risk factors of breast cancer among Palestinian in Southern area of the West Bank [master’s thesis]. Al-Quds University, Palestine, 2014.

Srour M, Almassri H, Almghari K: The relationship between serum levels of folic acid and developing of breast cancer among women in Gaza Strip (P05-033-19). *Curr Dev Nutr* 3:458, 2019 (suppl 1).

Yassin S, Younis M, Abuzerr S, et al: Extrinsic risk factors for women breast cancer in Gaza Strip, Palestine: Associations and interactions in a case-control study. *Adv Breast Cancer Res* 8:11-30, 2019.

One quantitative study on breast and colorectal cancer survival:

Panato C, Abusamaan K, Bidoli E, et al. Survival after the diagnosis of breast or colorectal cancer in the Gaza Strip from 2005 to 2014. *BMC Cancer* 18:632, 2018

One quantitative study of evaluation of diagnostic imaging services at nongovernmental health facilities:

Balousha M: Evaluation of diagnostic imaging services at nongovernmental organizations in Gaza Governorates [master’s thesis]. Al-Azhar University, Cairo, Egypt, 2018.

One study of diagnostic delay of breast cancer:

Al Shiekh, Alajerami YS, Etewa BB, et al: System delay in breast cancer diagnosis in Gaza Strip, Palestine. *J Oncol* 2019:5690938, 2019.

One study of awareness of risk factors and breast symptoms:

Elshami M, Kmeil HA, Abu-Jazar M, et al: Breast cancer awareness and barriers to early presentation in the Gaza Strip: A cross-sectional study. *JCO Glob Oncol* 10.1200/JGO.18.00095.

One study of a tumor marker for breast cancer:

Khattab B: Evaluation of plasma soluble human leukocyte antigen-G as a tumor marker for breast cancer patients in Gaza Strip [master’s thesis]. The Islamic University of Gaza, Gaza, Palestine, 2015.
